# A case of delirium in Tolosa-Hunt syndrome during corticosteroid therapy

**DOI:** 10.1186/s40981-018-0193-y

**Published:** 2018-07-31

**Authors:** Rie Ishikawa, Yuhei Ishikawa, Takeshi Kubota, Kumi Shirahama

**Affiliations:** 1 Department of Anesthesia and Pain Medicine, Hachinohe Heiwa Hospital, 4-6 Minatotakadai 2-chome, Hachinohe, 031-8545 Japan; 2 Department of Ophthalmology, Hachinohe Heiwa Hospital, 4-6 Minatotakadai 2-chome, Hachinohe, 031-8545 Japan

**Keywords:** Tolosa-Hunt syndrome, Corticosteroid, Delirium, Steroid psychosis

## Abstract

**Background:**

Corticosteroid therapy is useful for the resolution of pain and paresis in Tolosa-Hunt syndrome; however, there is no definitive protocol for appropriate dosing, route of administration, or duration of therapy. Steroid psychosis is an adverse reaction to corticosteroid therapy; in severe cases, it can develop into psychiatric disorders such as delirium, depression, and mania. In this case study, we report a patient with Tolosa-Hunt syndrome who developed delirium while receiving corticosteroid therapy.

**Case presentation:**

The patient was a 70-year-old man being treated for a main complaint of pain in the right eye accompanied by oculomotor paralysis. We suspected Tolosa-Hunt syndrome based on diagnostic imaging and other findings. Steroid pulse therapy was initiated with intravenous methylprednisolone at 1000 mg/day for 3 days, followed by oral prednisolone at 60 mg/day. The pain in the right eye disappeared the day after starting this regimen, and palpebral ptosis also improved. However, 5 days after starting treatment, the patient developed progressively worsening delirium, which was considered an adverse reaction to the steroid pulse therapy. Then, prednisolone treatment was temporarily suspended, and the delirium subsequently disappeared.

**Conclusions:**

The manifestation of steroid psychosis following corticosteroid therapy is dose dependent. Therefore, corticosteroid therapy for elderly patients requires caution and dose modulation because of likely adverse drug reactions.

## Background

Tolosa-Hunt syndrome (THS) is described in the International Classification of Headache Disorders (3rd edition) as unilateral orbital or periorbital pain associated with paresis of one or more of the third, fourth, and/or sixth cranial nerves caused by granulomatous inflammation in the cavernous sinus, superior orbital fissure, or orbit. The pain and paresis in THS resolve when adequately treated with corticosteroids [1]. However, previous reports showed that there is no definitive protocol for the dose, method, or duration of corticosteroid therapy in THS, and most of these factors depend on practice at the managing hospital or the judgment of the attending physician [2–8].

Although corticosteroids have a potent antiinflammatory effect, psychiatric symptoms can develop as an adverse reaction. Delirium, depression, mania, and other psychotic disorders reportedly develop as treatment-related neuropsychiatric symptoms [9].

Here, we report a patient with THS who was successfully treated by corticosteroid therapy, which induced severe delirium as an adverse reaction over the course of the treatment. We review the literature and address the issue of steroid dosing in THS therapy.

## Case presentation

The patient in this case study was a 70-year-old man (height 152 cm, weight 57 kg) with a history of hypertension who was receiving treatment from an ophthalmologist for pain in the right eye and blurred vision. He had been prescribed a course of loxoprofen sodium (60 mg), but the treatment was ineffective. He was referred to our hospital 5 days after symptom onset. In addition to the right eye pain and diplopia, findings during our initial examination included ipsilateral palpebral ptosis and oculomotor dysfunction, and oculomotor nerve paralysis was suspected. Simple cranial magnetic resonance imaging (MRI) revealed no intracranial space-occupying or periorbital lesions. The patient was prescribed tramadol hydrochloride (25 mg) for the eye pain, and the pain was relieved. However, the palpebral ptosis and diplopia persisted, and the patient was hospitalized for observation and treatment.

**Fig. 1:**
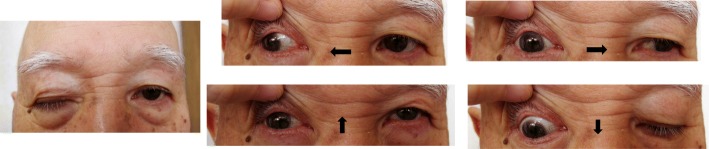
Neuro-ophthalmologic examination before treatment initiation shows right palpebral ptosis and paresis of the third right canal nerve

**Fig. 2:**
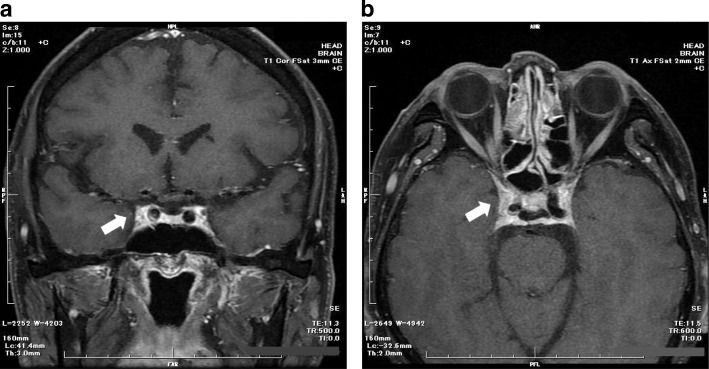
**a** Coronal T1 scan of brain MRI with gadolinium. **b** Axial T1 scan of orbital MRI with gadolinium. Both images show the lesion expanding from the right cavernous sinus to the orbital apex (arrows)

The findings at hospitalization are shown in Fig. 1. The patient showed limited lateral movement and elevation of the right eye in addition to palpebral ptosis but had no problems with visual acuity. Thus, he was diagnosed with incomplete oculomotor paralysis of the right eye. Cranial gadolinium-contrast MRI revealed slight expansion of the right cavernous sinus (relative to the contralateral site) but no clear signs of a tumor (Fig. 2). We ruled out diabetes-induced painful ophthalmoplegia, sarcoidosis, and other inflammatory diseases, as well as cerebrovascular disease based on the patient’s symptoms, physical examination, and imaging results. Considering the above findings, we suspected THS accompanied by oculomotor paralysis.

Sixteen days after symptom onset, the patient started steroid pulse therapy; he was treated with intravenous methylprednisolone at 1000 mg/day for 3 days. The patient developed hiccups, which are an adverse drug reaction to methylprednisolone, but this symptom disappeared after treatment with metoclopramide and shakuyakukanzoto, *a Chinese herbal medicine (**http://mpdb.nibiohn.go.jp/stork**).* The following day, the pain in the right eye disappeared, and the palpebral ptosis showed some improvement; however, the oculomotor dysfunction and diplopia persisted. The patient was started on a course of oral prednisolone at 60 mg/day subsequent to the steroid pulse therapy.

The patient developed progressively aggravated delirium at 5 days after the initiation of steroid pulse therapy. He showed clouding of consciousness accompanied by speech disturbance, abnormal sleep cycle, and marked appetite loss. No abnormalities were revealed on a cranial computed tomography scan performed that day, and the delirium was considered an adverse reaction to methylprednisolone. Accordingly, the oral prednisolone treatment (60 mg/day) was temporarily suspended. The delirium resolved subsequent to this suspension of treatment. By the day after prednisolone was suspended, the patient’s level of consciousness had recovered to the pretreatment level.

Two days after resolution of the delirium, oral prednisolone treatment was resumed at 10 mg/day. The patient manifested no subsequent hiccups, delirium, or disturbance of consciousness. The pain in the right eye completely disappeared, and palpebral ptosis, impaired right-eye elevation, and diplopia resolved. The prednisolone treatment at 10 mg/day was continued, and the patient was discharged from the hospital. After discharge, the oral prednisolone dose was tapered and then stopped at 53 days after the start of therapy. The patient showed no relapse after prednisolone cessation, and his treatment was regarded as complete. Follow-up MRI was not carried out since the patient smoothly recovered from the illness.

## Discussion

THS can be treated with corticosteroids, with the disappearance of eye pain within a number of days (approximately 1 to 4 days) and alleviation of ophthalmoplegia within a number of weeks (7 to 60 weeks) from the start of treatment [5, 8]. The patient in this case received intravenous methylprednisolone at 1000 mg/day for 3 days as steroid pulse therapy and then started receiving oral prednisolone at 60 mg/day. The eye pain disappeared, and oculomotor paralysis was resolved at 1 and 10 days after the start of methylprednisolone treatment, respectively. However, the patient developed hiccups and delirium as adverse reactions; thus, the treatment was temporarily suspended before its scheduled completion. Transient adverse reactions to steroid pulse therapy include dysgeusia (metallic taste), facial flushing, fever, and headache; however, the incidence of these adverse reactions is reportedly reduced with at least 1 h of intravenous administration. The patient in this case did not experience these adverse reactions. Other adverse reactions that may occur include arrhythmia, neuropsychiatric symptoms, triggering of latent infection, peptic ulcer, hyperglycemia, and osteoporosis.

Steroid psychosis can present with features ranging from mild mood changes to more serious signs such as multiple memory deficits, overt mood changes (depression, mania), psychiatric symptoms, and delirium as the condition becomes more severe [10]. The relevant incidence reportedly ranges from 70 to 80% for mood disorders such as mania and depression; 10% for schizophrenia-type conditions such as confusion, hallucinations, and thought disorders; and approximately 10% for delirium. Delirium is common in older patients and progresses to disturbed consciousness; however, recent reports have emphasized that slight, subdelirious cognitive dysfunction—particularly recurrent memory loss—can frequently develop as a result of corticosteroid therapy [11].

The mechanism of steroid psychosis remains unclear, and psychiatric symptoms can emerge as quickly as 3 to 5 days from the start of corticosteroid therapy [12]. Psychiatric symptoms disappear in 50 and 90% of cases by 1 and 6 weeks after suspension of corticosteroid therapy, respectively, and all patients ultimately return to a normal condition [9]. The patient in this case manifested delirium at 4 days after the start of steroid pulse therapy, and the delirium disappeared shortly after the suspension of corticosteroid therapy; his progress was consistent with the above report [9]. Withdrawal symptoms can occur after sudden suspension of corticosteroid therapy, which requires attention, but the patient in this case developed no such symptoms. At 2 days after corticosteroid suspension, therapy was resumed with low-dose oral administration, and the patient showed smooth progress thereafter.

Corticosteroid psychosis develops in a dose-dependent manner. Specifically, its incidence with prednisolone therapy is 1.3% at ≤ 40 mg/day, 4.6% at 40–80 mg/day, and 18.4% at ≥ 80 mg/day; 59.5 mg/day is the reported mean dose at which patients manifest psychiatric symptoms [13]. Lewis et al. supported these dose-dependent effects; 23% of patients receiving less than 40 mg/day prednisolone developed psychiatric symptoms, whereas 77% of patients receiving greater than 40 mg/day developed these symptoms [9]. Although a straightforward dose-response effect on psychiatric adverse reactions has not yet been elucidated, these reports suggest that a prednisolone dose less than 40 mg/day might safely prevent these reactions. Steroid therapy for THS currently lacks any protocol-specified dosage; accordingly, corticosteroid therapy involves varied doses, methods, and durations (Table 1) [2–8]. An initial prednisolone dose of 1.0 to 1.5 mg/kg/day is commonly found in available reports. However, the therapeutic effect reportedly does not differ between doses above and below 0.5 mg/kg/day [14]. The patient in this case was an elderly man of small stature; consequently, there were concerns over adverse reactions to corticosteroid therapy, and dose modulation was required. Specifically, in this case, oral prednisolone should have been initiated at 30 mg/day (0.5 mg/kg/day) without intravenous administration of methylprednisolone. We were able to proceed with dose tapering followed by therapy cessation while monitoring the patient’s condition.

**Table 1:** Case reports involving patients who were given corticosteroid therapy

Author (year)	Steroid	Initial dose	Dosing period	Route of initial administration	Tapering period
Mombaerts I (1996) [2]	Corticosteroid	60 mg/day	2 weeks	PO	Several months
Cakirer S (2003) [3]	Prednisolone	80 mg/day	2 weeks	IV	6 weeks
Colnaghi S (2006) [4]	Prednisolone	75 mg/day	4 months	–	–
Curone M (2009) [5]	Prednisolone	1–1.5 mg/kg/day	–	PO	5–6 weeks
Schuknecht B (2009) [6]	Prednisolone	1–1.5 mg/kg/day	–	PO	– Reduced based on clinical response
İlgen Uslu F (2015) [7]	Methylprednisolone	1 g IV 5 days 60 mg PO 1 week (1 mg/kg/day)	1 week	IV	2 months
Hao R (2015) [8]	Dexamethasone	10–15 mg/day	3 days	IV	Until signs and lesion disappeared

*–* not described, *PO* per os, *IV* intravenous injection

The patient was diagnosed with THS based on his symptoms, imaging findings, and treatment response. Diagnosing THS is often beset with difficulties, although the diagnosis is based on the criteria stated in the International Classification of Headache Disorders (3rd edition). Imaging findings are crucial for judgments made with these diagnostic criteria. In patients with clinical THS who underwent MRI scans, Colnaghi et al. identified positive findings in 92.1% of cases, and characteristic lesions were not detectable in 7.9% of cases [15]. Thus, comparing pre- and postcorticosteroid therapy MRI findings is considered important for diagnosis and improves detection rates [3]. Postcorticosteroid therapy MRI scans enable the verification of the reduced size of granulomatous lesions. In this case, MRI revealed expansion of the right cavernous sinus, although the presence of a granulomatous lesion could not be clearly identified. Ideally, we should have performed MRI scans again to verify the normal-sized right cavernous sinus. However, we considered follow-up MRI scans unnecessary because of the smooth recovery from the illness in the present case.

In the present case, steroid pulse therapy was effective but induced delirium. Therefore, corticosteroid therapy for elderly patients requires careful symptom monitoring and dose modulation to avoid serious adverse reactions.

## Abbreviations

IgG4-RD: IgG4-related disease
MRI: Magnetic resonance imaging
THS: Tolosa-Hunt syndrome
